# Efficient quantum random number generation via simultaneously detecting photons in temporal and spatial dimensions

**DOI:** 10.1038/s41598-025-03680-7

**Published:** 2025-05-30

**Authors:** Bingkun Wang, Jianyong Hu, Xingjian Li, Jianqiang Liu, Shuxiao Wu, Haizhen Li, Liwen Zhang, Changgang Yang, Zhixing Qiao, Ruiyun Chen, Guofeng Zhang, Chengbing Qin, Liantuan Xiao, Suotang Jia

**Affiliations:** 1 State Key Laboratory of Quantum Optics Technologies and Devices, Institute of Laser Spectroscopy, Shanxi University, Taiyuan, 030006 China; 2 Collaborative Innovation Center of Extreme Optics, Shanxi University, Taiyuan, 030006 China; 3 Hefei National Laboratory, Hefei, 230088 China; 4 College of Information Engineering, Shanxi Vocational University of Engineering Science and Technology, Jinzhong, 030619 China; 5 School of Physics and Information Engineering, Shanxi Normal University, Taiyuan, 030031 China; 6 College of Medical Imaging, Shanxi Medical University, Taiyuan, 030001 China; 7 College of Physics, Taiyuan University of Technology, Taiyuan, 030600 China

**Keywords:** Optical techniques, Quantum physics, Qubits

## Abstract

Quantum random number generators (QRNGs) produce true random numbers with significant applications in quantum communication and numerical computation, where high-rate random number generation is critical. Photon detection-based quantum random-number generation methods have been widely studied. However, the generation rate is constrained by the count rate of single-photon detectors. This study proposes an efficient method that enhances random number generation by simultaneously detecting photons in temporal and spatial dimensions. We achieved simultaneous detection of photon arrival time and spatial position by employing a laboratory-developed 5 × 5 single-photon detector array and a high-saturation count rate multichannel time-to-digital converter. The maximum efficiency of the method was 21.1 bits per event and it maintained a consistent efficiency of 17.6 bits per event while achieving a random number generation rate of 2.1 Gbps. The proposed QRNG approach offers a promising pathway for significantly increasing random number generation rates, benefiting applications that require secure and high-speed random number sequences.

Random number generators are essential in fields such as cryptography, numerical computation, and blockchain technology^1^. However, conventional pseudorandom number-generators (PRNGs) produce sequences with predictable determinism and limited periodicity, which can’t meet requirement of some applications, especially in high-security applications like quantum key distribution (QKD)^2,3^. It underscores the importance of addressing potential security vulnerabilities. The uncertainty principle in quantum mechanics offers an ideal physical basis for generating true random numbers, and the security under untrusted sources has also been validated^4,5^.

Quantum random number generators (QRNGs) have been proposed using various physical systems, including atomic systems^6^, electronic system^7^, radioactive decay^8^, and photon detection^9–11^. Among these, photon-detection-based QRNGs have gained attention due to their straightforward design^12^. A fundamental approach involves directing photons through a beam splitter or a polarizing beam splitter and measuring the collapse of their spatial paths to produce classical random bits^13–15^.

In earlier studies, only one random bit could be extracted from one detection event^16^. Enhancing the efficiency of random number generation per detection event has become a key strategy for boosting generation rates because the saturation photon count rate of single-photon detectors (SPD) cannot be infinitely increased. Multipath measurement strategies have been proposed to address the efficiency bottleneck^17^. Gräfe increased random number efficiency to 4 bits/event by preparing single-photon W-states with 16 spatial modes^18^. Yan et al. achieved 16 bits/event by extracting multi-bit random numbers from the positional coordinates of each detected photon using a 256 × 256 pixel SPD array^19^. However, further increasing the pixel scale of SPD arrays yields diminishing returns; for instance, expanding from a 256 × 256 to a 512 × 512 array only increases efficiency by 2 bits/event.

Another efficient method for quantum random number generation involves measuring the collapse of coherent photons in the temporal dimension^20–22^. Nie achieved 5.5 bits/event using an external periodic reference to obtain uniformly distributed raw statistical data^23^. Wahl attained 16 bits/event by analyzing time intervals between photon arrivals and applying a postprocessing method known as “resilient function” to convert the resulting exponential distribution data^24^. Time and spatial dimension measurements can be conducted simultaneously, significantly enhancing random number generation efficiency^25,26^. The joint measurement of time and spatial dimensions was performed in Ref^27^; however, efficiency remained low due to the focus on comparing waiting time differences. Lin measured time and spatial distributions of dark counts using a multichannel silicon photomultiplier array. This method does not involve a light source. However, it generates random numbers from electronic thermal noise, with a random number generation rate of only 63.54 Mbps^28^.

This study proposes a method to generate high-efficiency quantum random numbers that leverages joint temporal and spatial measurements of coherent photons, enhancing the efficiency and rate of random number generation. We successfully achieved simultaneous detection of photon arrival time and spatial position using a laboratory-developed 5 × 5 SPD array and a high-saturation count rate multichannel time-to-digital converter (TDC). The method achieved a random number generation efficiency and generation rate of 21.1 bits/event and 2.1 Gbps, respectively, fully exploiting the temporal and spatial coherence of coherent-state photons, providing a new pathway for high-rate quantum random number generation.

Randomness in photon-detection-based quantum random number generation originates from the consumption of coherence. We increased the random-number generation efficiency per single-photon detection event by detecting the temporal and spatial dimensions of photons, thereby increasing the overall generation rate.

Theoretically, an infinite number of random bits can be extracted from a single photon upon detection in the temporal dimension. However, practical operations face limitations primarily due to the true time resolution of the TDC, which determines the number of extractable random bits per detected photon. Additionally, the maximum photon count rate of an SPD is constrained by dead time. We used a self-developed SPD array for detection. Photon counts of coherent states follow a Poisson distribution, and the time intervals *Δt* between consecutive photon detections in temporal measurements follow an exponential distribution^21^. Considering that *Δt* is discretized in practical systems, particularly due to the finite time resolution *δt* of the TDC, it is more appropriate to describe the photon detection statistics using a discrete model. The center of the *j*^*th*^ bin is denoted by *Δt*_*j*_ = *j* ⋅ *δt*. The time interval distribution is:

1 $$P_{{(\Delta t_{j} )}} = \left\{ {\begin{array}{*{20}l} 0 \hfill & {\Delta t_{j} < \tau _{s} } \hfill \\ {\lambda \cdot \delta t \cdot e^{{ - \lambda (\Delta t_{j} - \tau _{s} )}} } \hfill & {\Delta t_{j} > \tau _{s} } \hfill \\ \end{array} } \right.$$

where *λ* represents the mean photon count rate per second and *τ*_*s*_ denotes the dead time of the SPD.

Consider an SPD array with *N* pixels. One pixel enters a dead period and transitions to an “off” state after photon detection, reducing the number of active pixels to *N-1* and diminishing overall detection efficiency. Several pixels will enter “off” states if multiple detectors capture photons simultaneously, limiting the probability of photon detection at that moment. Consequently, the distribution of photon detection time intervals becomes:

2 $$P_{{(\Delta t_{j} )}} = \left\{ {\begin{array}{*{20}l} {\frac{{N - \lambda _{s} }}{N}\lambda \cdot \delta t \cdot e^{{ - \lambda \Delta t_{j} }} } \hfill & {\Delta t_{j} < \tau _{s} } \hfill \\ {\lambda \cdot \delta t \cdot e^{{ - \lambda (\Delta t_{j} - \tau _{s} )}} } \hfill & {\Delta t_{j} > \tau _{s} } \hfill \\ \end{array} } \right.$$

where *λ*_*s*_​ = *λ* ⋅ *τ*_*s*_ represents the average number of photons per second that arrive during the detector’s dead time *τ*_*s*_ and are hence unrecorded. When the SPD array contains a sufficient number of pixels and the photon count rate is low, the distribution of detection time intervals described by Eq. (2) follows a truncated exponential distribution, with exponential distributions before and after the dead time *τ*_*s*_, where the dead time causes the overall distribution to be truncated at *τ*_*s*_. We can derive the information entropy of a photon detection event from Eq. (2), representing the maximum number of random bits extractable from such an event:

3 $$H_{{time}} = - \sum\limits_{{j = 1}}^{\infty } {P_{{\left( {\Delta t_{j} } \right)}}^{{}} \cdot \log {}_{2}(P_{{\left( {\Delta t_{j} } \right)}}^{{}} )}$$

where *Δt*_*j*_ ​ represents the *j-th* discrete time interval with resolution *δ*_*t*_, and the distribution *P(Δt*_*j*_*)* is defined over the set of all possible time differences. In theory, the summation in Eq. (3) extends to infinity, following the standard definition of discrete Shannon entropy. However, in practical implementation, the distribution is estimated based on a finite number of observed time intervals. Specifically, the summation is truncated at the maximum observable time difference *Δt*_*max*_ ​, and the number of discrete bins is given by *M = Δt*_*max*_ */ δt*. Beyond this point, the probabilities are effectively zero due to limited measurement duration and resolution. This truncation yields a good approximation without significantly affecting the computed entropy. It should be noted that Eq. (3) represents an upper bound, while in reality the actual extractable entropy is further limited by the efficiency of the processing algorithm, and also the crosstalk and dead time of the detectors.

The number of random bits extractable per photon, considering spatial dimension detection, correlates with the scale of the detector array. However, the possibility that some detector pixels may be in an “off” state must also be considered. If all detectors are “on,” a photon arriving can be detected by any of the N detectors. Assuming each detector has an equal probability of *1/N* of detecting a photon, the maximum number of random bits extractable from a photon detection event is log_2_^*(N)*^. However, if *k* detectors are simultaneously “off,” only *N-k* detectors can detect the photon, reducing the maximum number of random bits to log_2_^*(N-k)*^. The number of detectors in the “on” state can be expressed as a function of the photon count rate:

4 $$N_{{on}} = \sum\limits_{{k = 0}}^{N} {\left( {N - k} \right)\frac{{\lambda _{s} ^{k} e^{{ - \lambda _{s} }} }}{{k!}}},$$

Hence, the corresponding information entropy for a one-photon detection event in the spatial dimension is:

5 $${H_{space}}={\log _2}({N_{on}}) \leqslant {\log _2}(N),$$

The intervals between photon arrivals decrease as the photon count rate increases, increasing the likelihood of detectors becoming inoperable due to dead time. This reduces the number of available spatial channels for photon selection, decreasing the spatial entropy of the system. The equality in Eq. (5) can be approximated only at low photon count rates.

We focus on achieving a uniform probability distribution to maximize the randomness. Therefore, raw data must undergo “whitening” to eliminate biases introduced by exponential distributions and experimental system factors. These biases may stem from equipment flaws such as multiphoton emissions, dead times, dark counts, after-pulses, uneven detector efficiency, and uneven light intensity distribution. We employed a Toplitz matrix hash function, accelerated by FFT, to process the raw data, mapping it onto a more uniformly distributed sequence of random bits through its hashing properties^29–31^.

This study used an SPD array with time-resolution capabilities, simultaneously extracting random numbers from temporal and spatial dimensions for each photon detection event. Given a photon count rate *λ*, the random number generation rate in our system is calculated as follows:

6 $$R = \lambda \cdot \left( {H_{{time}} + H_{{space}} } \right)$$

However, increasing the photon count rate may reduce both *H*_*time*_ and *H*_*space*_. Therefore, a higher photon count rate does not necessarily lead to a higher random number generation rate. The formulas provided are meant to serve as theoretical references, yet practical implementation must take into account the efficiency of the algorithm. In our work, these formulas not only established a rigorous theoretical basis but also guided the concrete realization of the algorithm.

An efficient QRNG was developed based on the joint measurement of time and space (see Fig. 1). The experimental system included a light source, an adjustable optical attenuator, a 1 × 25 fiber beam splitter, a 5 × 5 SPD array, and a TDC. The light source was a single-frequency narrow-linewidth laser (PL-NL-633-30-A81-PA) with a wavelength of 633 nm. The laser current output was adjusted to control the desired output power. The photon count rate was precisely regulated using an adjustable optical attenuator.

**Fig. 1:**
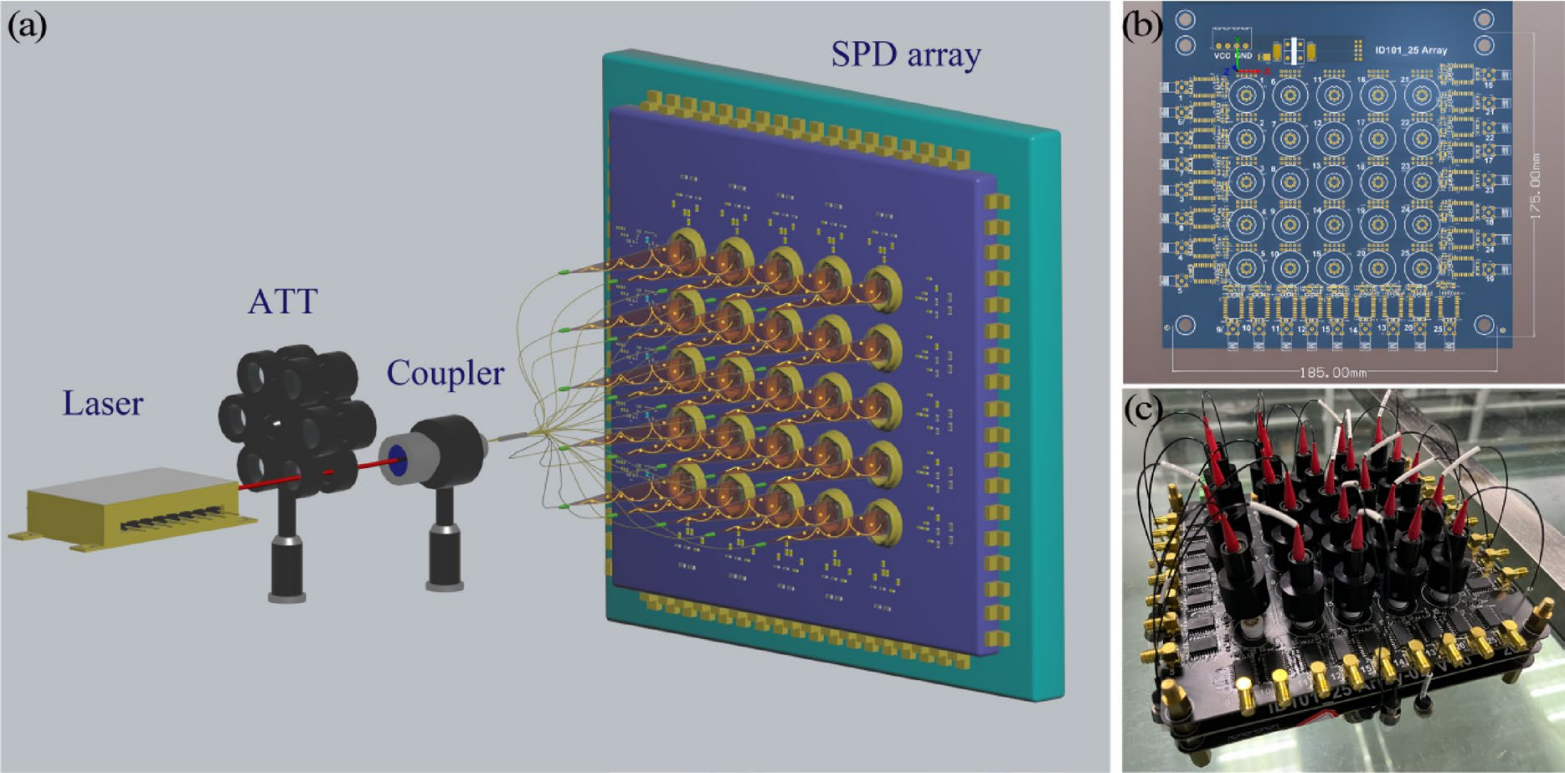
Efficient quantum random number generator. (**a**) Schematic of the experimental setup. (**b**) Drive circuit for the 5 × 5 detector array. (**c**) 5 × 5 SPD array.

After that, the photon was input into a 1 × 25 beam splitter, following the quantum mechanical principle of superposition. The photon existed simultaneously in 25 paths and collapsed into one of these paths upon detection. The SPD array was a laboratory-developed 5 × 5 silicon single-photon avalanche diode (iD101-50) with integrated power supply, temperature control, and pulse screening and shaping modules. The experiments were conducted in a room-temperature environment. Although the SPAD module includes a TEC cooler that maintains the detector at  – 34 °C, this temperature control is internal to the device and is standard for ensuring optimal detector performance. We did not encounter any thermal instability during the measurements. The SPD exhibited dead time, dark count rate, after-pulse probability, and detection efficiency of 45 ns, ~ 100 counts per second (cps), 0.5%, and 25% (at 633 nm), respectively. The pulse arrival time of the array detector was recorded using a self-developed high-saturation count-rate multichannel TDC, featuring a saturation count rate of 200 Mcps and a time bin size of 1 ps. The maximum number of extractable random bits per event is primarily determined by the RMS resolution of the TDC. Our TDC exhibits an RMS timing resolution of approximately 23.8 ps, which defines the fundamental time measurement precision of our system. Temporal and spatial information from photon detection events was extracted using the time and channel labels of the TDC, respectively. The final random number sequence was generated using a hash function.

The distribution of photon arrival time intervals was analyzed at different photon count rates. The count rate we refer to is the sum of all channels. To simulate this process, we start with parameter initialization, and first set the detector dead time *τ*_*s*_, the total input photon count rate *λ’* of the system, and the 1-second sampling time window. Subsequently, raw photon arrival time sequences obeying a uniform distribution are generated independently for each detection channel. For the original sequence of each channel, the time is first arranged in ascending order, and then the iterative screening is performed: the time interval of neighboring photons is calculated, and if the interval is less than or equal to the dead time, the latter photon is rejected, and the process is repeated until all the time difference is greater than *τ*_*s*_ to form the corrected sequence. After completing all the channel corrections, the valid photon events of each channel are merged, and the merged sequence is sorted twice to ensure the global timing consistency. Then, the time difference of neighboring photons is calculated for the sorted total sequence. Statistical distribution after data segmentation in 1 ps interval steps to generate histograms of time-difference distributions and probability density functions to accurately characterize the output timing of the array detector. We plot this flowchart in Fig. 2b. As the photon count rate increased, its statistical distribution became more concentrated in regions of shorter time intervals, leading to a more imbalanced probability distribution. This trend is illustrated in Fig. 2, where darker lines represent theoretical simulations and lighter scattered points correspond to experimental data. This imbalance reduced the efficiency of random number generation for individual photon detection events (see Eq. 3). The efficiencies of random number generation based on time dimension measurements at photon count rates of 6.8, 31.0, 62.0, and 121.0 Mcps were 21.1, 19.9, 18.8, and 17.6 bits/event, respectively. The statistical distribution results also indicate a deviation from a standard exponential distribution (see Eq. 2). The inflection point caused by the dead time of the detector became indistinct at high photon count rates due to the simultaneous occurrence of multiple photon detection events, averaging the conditional probability distribution. The inflection point was due to the dead time of the detector, which was not visible at the photon count rate of 121.0 Mcps.

**Fig. 2:**
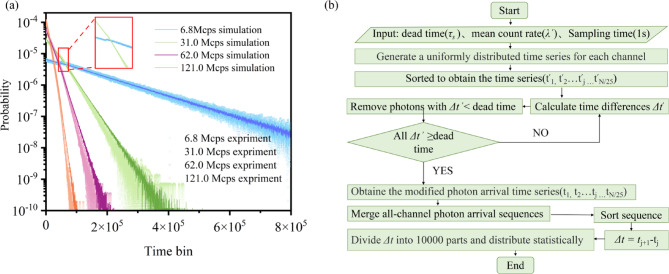
(**a**) Distribution of photon arrival time intervals at different photon count rates (6.8, 31.0, 62.0, 121.0 Mcps). Darker lines represent theoretical simulations, while lighter scattered points denote experimental data. Both the simulated and experimental data exhibit an exponentially decaying distribution trend; (**b**) The flow chart of the simulation program.

The spatial distribution of random numbers must be uniform. However, the observed distribution demonstrates inhomogeneity due to hardware limitations and operational factors (see Fig. 3a). The inhomogeneity arises from two primary factors. First, the non-uniformity intensity distribution of the beam entering the beam splitter leads to varying probabilities of photon detection by each detector. Second, achieving uniform detection efficiency across all single-photon detection is challenging due to its bi-stochastic Poisson point process nature. Consequently, differences in photon count rates among detectors exacerbate spatial distribution heterogeneity, degrading random number generation efficiency. Hence, an efficient FFT-Toeplitz hashing function was employed to process the raw data and generate a reliable sequence of true random bits.

**Fig. 3:**
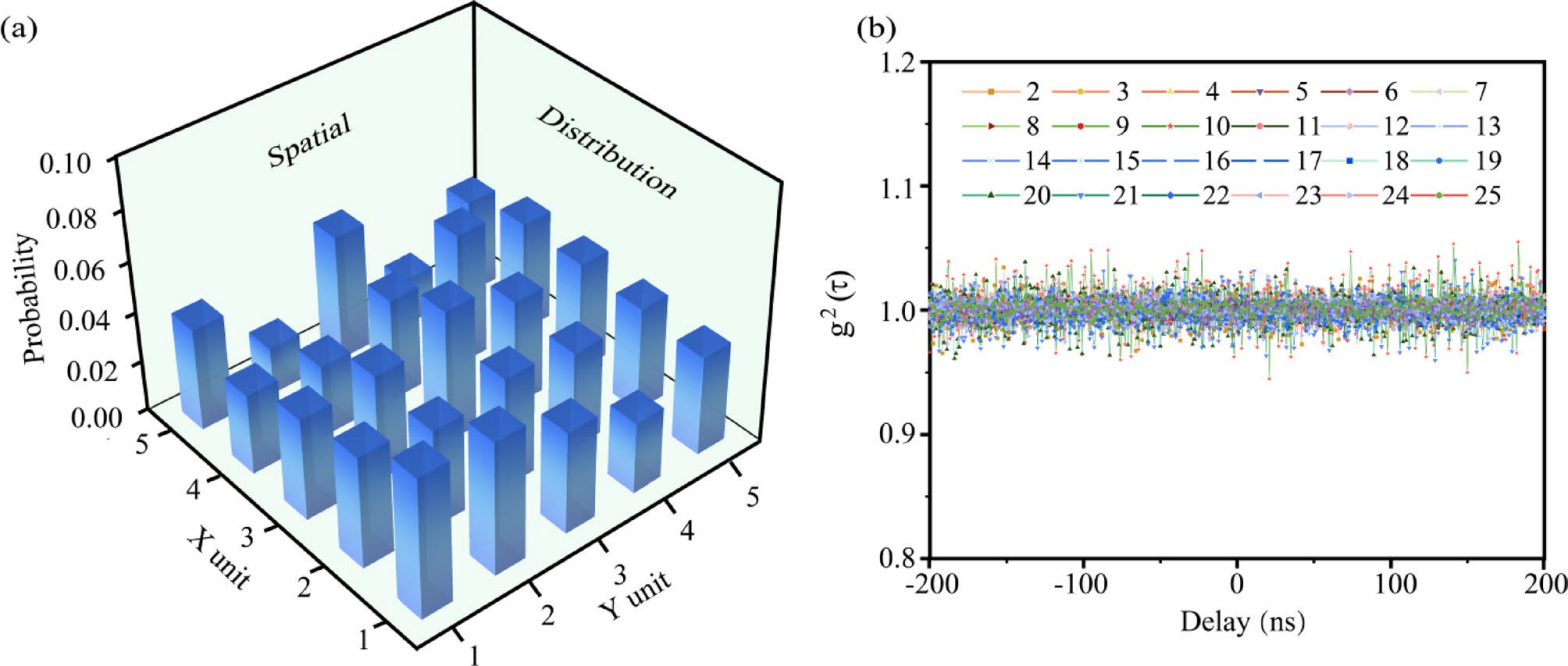
(**a**) Spatial distribution of photon detection probability for the 5 × 5 SPD array. (**b**) Second-order correlation function between each SPD, with the horizontal axis representing the time delay, ranging from  – 200 ns to 200 ns.

Increased photon count rates raise concerns about potential correlations in photon detection between adjacent SPDs, which may affect data randomness. Therefore, we performed a second-order correlation analysis between a fixed SPD and other SPDs to quantify these correlations. The second order correlation coefficients between adjacent SPDs were found to be very close to one (ranging from 0.95 to 1.05), indicating that the differences in photon detection between SPDs are minimal and do not show significant correlations. (Fig. 3b), indicating the absence of a significant correlation between adjacent SPDs. This result confirms the independence of photon detection events for each SPD, ensuring the randomness of the experimental dataset.

**Table 1:** The simulated spatial random bits/event, the experimental spatial random bits/event, the simulated temporal random bits/event and the experimental temporal random bits/event at different counting rates.

Photon count rates (Mcps)	Simulated spatial (bits/event)	Experimental spatial (bits/event)	Simulated temporal (bits/event)	Experimental temporal (bits/event)
6.8	6	5.78	17.7	15.34
20.5	5.93	5.73	15.58	14.67
31.0	5.89	5.69	14.98	14.21
37.2	5.86	5.66	14.71	13.93
51.0	5.81	5.61	14.26	13.53
62.0	5.77	5.56	13.99	13.25
73.0	5.72	5.52	13.73	13.02
88.3	5.65	5.45	13.49	12.75
100.4	5.60	5.4	13.28	12.58
121.0	5.50	5.3	13.05	12.32

The Raw data were “whitened” using the FFT-Toeplitz-Hash function to obtain the final random bit sequence. We present the simulated and experimentally obtained per-event bit efficiencies for both Temporal and Spatial information in detail in Table 1. Figure 4 shows the total simulated number of random Bits generated per photon detection event (green solid line) and the number of total Bits extracted at different photon count rates (green dotted line). the maximum efficiency of the random number generation achieved was 21.1 bits/event. We take this data as an example to analyze the whitening efficiency, which is 28.3 bits/event before whitening and 21.1 bits/event after whitening, that is, 7.2 bits per event is lost, and the loss rate is about 25.4%. This proves the efficiency of our hash processing, which preserves most of the randomness of the original data. As we mentioned earlier that the entropy of the theory gives an upper bound, in practice the efficiency of the algorithm also has an impact on the result. the random number generation rate increased significantly with the increase in the photon count rate. the random-number generation efficiency at a photon count rate of 121.0 Mcps was 17.6 bits/event, and the generation rate reached 2.1 Gbps (see Fig. 4a). In addition we estimate the quality of randomness using the minimum entropy (*H*_min_) of bits after the FFT-Toeplitz hashing. the results for each count rate are listed in Table 2.

**Table 2:** Minimum entropy of random bit distribution after FFT-Toeplitz hashing at different count rates.

Photon count rates (Mcps)	6.8	20.5	31.0	37.2	51.0
*H*_min_​	0.99983	0.99995	0.99996	0.99999	1.00000
Photon count rates (Mcps)	62.0	73.0	88.3	100.4	121.0
*H*_min_​	0.99988	1.00000	0.99999	0.99993	0.99998

The randomness of the generated random numbers was verified using the NIST statistical test suite^32^. A total of 20 independent random bit sequences were tested, corresponding to the bits extracted from spatial and temporal correlations at ten different count rates. All test items in the suite were successfully passed, with each p-value exceeding 0.01 and an overall pass ratio greater than 0.98, indicating favorable statistical performance. The values shown in Fig. 4b represent the average results across these 20 sequences. To further validate the quality of randomness, the distribution of p-values obtained from the NIST tests was analyzed using the Kolmogorov-Smirnov (K-S) test. The results confirmed that the p-values were uniformly distributed, supporting the hypothesis that the bit sequences exhibit true randomness. Specifically, for each of the 15 statistical tests within the NIST suite, the average p-values across the 20 samples were subjected to the K-S test, and all yielded p-values greater than 0.05, thus satisfying the conditions for uniformity. We also clarify that in the context of statistical hypothesis testing, the absolute value of a p-value (e.g., 0.01 vs. 0.99) does not by itself indicate better or worse randomness. Instead, a uniformly distributed set of p-values across many tests and samples serves as a stronger indicator of high-quality randomness.

**Fig. 4:**
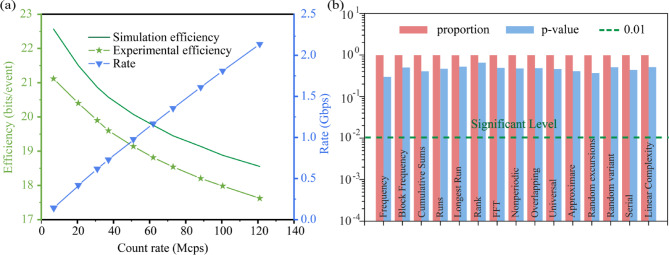
(**a**) Efficiency (green curve) and rate (blue curve) of random number generation for various photon count rates. (**b**) Randomness test.

In conclude, this study proposes an efficient method for quantum random number generation by simultaneously detecting coherent photons in both temporal and spatial dimensions. We employed a laboratory-developed multichannel SPD array and a high-saturation count-rate multichannel TDC to perform precise photon measurements across both domains. The maximum efficiency of the random number generation was 21.1 bits/event. The method maintained a consistent efficiency of 17.6 bits/event while achieving a random number generation rate of 2.1 Gbps. All experimental results met the NIST standard for randomness testing. The proposed method offers a novel approach for high-rate random number generation.

## Data Availability

The datasets generated and analysed during the current study are available from the corresponding author on reasonable request.
